# The Roles of IRF-8 in Regulating IL-9-Mediated Immunologic Mechanisms in the Development of DLBCL: A State-of-the-Art Literature Review

**DOI:** 10.3389/fonc.2022.817069

**Published:** 2022-02-08

**Authors:** Mingyue Cai, Na Chen

**Affiliations:** ^1^ Provincial Hospital Affiliated to Shandong First Medical University, Department of Hematology, Jinan, China; ^2^ School of Medicine, Shandong University, Jinan, China

**Keywords:** IRF-8, IL-9, AP-1 family, diffuse large B-cell lymphoma, immunologic mechanism

## Abstract

Interferon regulatory factor 8 (IRF-8) is a transcription suppressor that functions through associations with other transcription factors, contributing to the growth and differentiation of bone marrow cells and the activation of macrophages. IRF-8 expression profoundly affects pathogenic processes ranging from infections to blood diseases. Interleukin-9 (IL-9) is a multipotent cytokine that acts on a variety of immune cells by binding to the IL-9 receptor (IL-9R) and is involved in a variety of diseases such as cancer, autoimmune diseases, and other pathogen-mediated immune regulatory diseases. Studies have shown that IL-9 levels are significantly increased in the serum of patients with diffuse large B-cell lymphoma (DLBCL), and IL-9 levels are correlated with the DLBCL prognostic index. The activator protein-1 (AP-1) complex is a dimeric transcription factor that plays a critical role in cellular proliferation, apoptosis, angiogenesis, oncogene-induced transformation, and invasion by controlling basic and induced transcription of several genes containing the AP-1 locus. The AP-1 complex is involved in many cancers, including hematological tumors. In this report, we systematically review the precise roles of IL-9, IRF-8, and AP-1 in tumor development, particularly with regard to DLBCL. Finally, the recent progress in IRF-8 and IL-9 research is presented; the possible relationship among IRF-8, IL-9, and AP-1 family members is analyzed; and future research prospects are discussed.

## Introduction

Diffuse large B-cell lymphoma (DLBCL) is the most common lymphoid malignancy in adults, and it was defined as a diffuse growth of neoplastic large B lymphoid cells with a nuclear size equal to or exceeding normal macrophage nuclei by the World Health Organization. DLBCL that could not be classified into a specific entity are diagnosed as unspecified DLBCL. DLBCL is the most common type of lymphoma, accounting for about 25-35% of all non-Hodgkin lymphoma (NHL). Several phase III clinical trials have presented the treatment regimen of rituximab, cyclophosphamide, adriamycin, vincristine, and prednisone (R-CHOP regimen) for DLBCL patients, and within 50-70% of DLBCL patients have been successfully treated with R-CHOP regimen. However, R-CHOP failures are principally due to either primary refractoriness or relapse after reaching a complete response (CR). Salvage chemotherapy followed by autologous stem cell transplantation (ASCT) remain the standard of care for only 10% refractory or recurrent DLBCL patients, indicating that the treatment requirements have not been fully met yet. Therefore, investigation of the molecular mechanism of DLBCL is clinically significant for individualized treatment of DLBCL patients. Interferon regulatory factor-8 (IRF-8) plays a regulatory role in the differentiation of TH17 cells, macrophages, and dendritic cells (DCs). Activator protein-1 (AP-1) could enhance the interactions of IRF-8 with other signaling molecules, while the exact roles of IL-9 and IRF-8 in DLBCL, as well as their relationships with AP-1 family members have still remained elusive. Previous studies have demonstrated that the development and progression of DLBCL are associated with abnormal expressions of various factors. Therefore, further in-depth researches on specific biomarkers, signaling pathways, and pathogenesis of DLBCL need to be conducted to identify further effective targeted therapies for DLBCL patients.

## IRF-8

Interferon regulatory factor 8 (IRF-8), also known as interferon consensus sequence-binding protein, is a component of the IRF transcription factor family (1–3).

### The Amino Acid Sequences of IRF-8 and IRF-4 Have High Similarity and Play a Synergistic Role in Tumor Progression

Of all the IRF family members, IRF-4 and IRF-8 have the highest amino acid sequence similarity, and both could interact with some common chaperones (e.g., PU.1 and E47) to regulate a group of overlapped target promoters (4–7). IRF-4 (-) was found with a progressive systemic lymph node enlargement and a severe deficiency of functions of B and T lymphocytes (8). IRF-8 could also bind to PU.1 and stimulate the activity of ETS/IRF composite element (EICE) (9–15), and the interaction with IRF-1/2 and PU.1 could substantially enhance the binding activity of IRF-8 to DNA (11). PU.1 could also interact with IRF-4 to activate transcription through EICE (4). The transcription factor PU.1 is one of the proteins that directly interacts with IRF-8, which then regulates the expression levels of various myeloid-specific genes (16). PU.1 and IRF-8 could synergistically activate the promoters of various myeloid-specific genes through EICE, and thus, PU.1 has been proven to be a definite candidate partner of IRF-8 in regulating the development of myeloid cells. Interferon regulatory factor-4 (IRF-4) functions as a tumor suppressive factor in both B and T cell lineages (17, 18). The capabilities of IRF-4 in transforming lymphocytes, the abnormal expression modes in B- and T-cell lymphomas, and leukemia have already been documented (19, 20). Besides, IRF-8 is a critical regulator of myelopoiesis (21). Chronic myeloid leukemia (CML)-like diseases were observed in IRF-8-deficient mice at the age of 10-16 weeks, and about one third of mice were associated with myeloid and lymphatic systems, and then died at the age of 50 weeks (22–24). Several studies have demonstrated that the mechanisms involved in tumor-suppressing effects of IRF-8 include the downregulation of *Bcl-2* expression, as well as the upregulation of c-Myc inhibitor, blimp1, and METs (17). During B cell development, IRF-4/8 double deficiencies could prevent the transition of large cycling pre-B cells to small resting pre-B cells (25, 26). The clonal proliferation of pre-B cells would continue in the deficiencies of IRF-4/8, consequently increasing the risk of malignant transition of cells. Mice with both IRF-4 and IRF-8 deficiencies could develop CML-like diseases with higher invasiveness than mice with only IRF-8 deficiency. All mice with deficiencies of IRF-4 and IRF-8 finally developed B lymphoblastic leukemia/lymphoma at the age of 25 weeks, and then died. These findings demonstrated that deficiencies of IRF-4 and IRF-8 have synergistic effects in regulating the development and progression of bone marrow tumors and lymphomas (27).

### IRF-8 (-) Leads to Macrophage Immune Deficiency and CML-Like Disease

IRF-8 promotes the differentiation of myeloid progenitor cells to macrophages, while it inhibits the differentiation to granulocytes. IRF-8(-) mice have a higher number of bone marrow-derived progenitor cells (BMPCs), which are preferentially differentiated to granulocytes, whereas they cannot be effectively differentiated to macrophages. IRF8(-) mice are accompanied with immunodeficiency, and are also sensitive to various pathogens. In addition, macrophages in IRF8(-) mice are defected in various functions, including capabilities in inducing *IL-12 p40* and some IFN-γ-responsive *genes*. IRF-8 can regulate the induction of *IL-12 p40 gene*, thereby significantly influencing the differentiation of natural killer (NK) and Th1 cells in an IFN-γ-dependent manner (22, 28, 29). IL-12 could regulate the production of NK and CD4+ T cells. In turn, IFN-γ could induce the expressions of effectors, inducing potent anti-viral, anti-toxic, and anti-parasitic activities. IRF-8 alone could activate the promoter of *IL-12 p40* in mice and humans, and consequently promote the IFN-γ-dependent resistance. In addition, IFN-γ-induced IRF-8 is the major activator of *IL-12 p40* in macrophages. Thus, IFN-γ, as a macrophage-activating factor, can stimulate numerous genes in macrophages to induce the activities of macrophages, such as phagocytic effects, anti-bacterial effects, cytokine production, and antigen presentation, and it has been considered as the basis for the defense of host against infection (17). IRF-8 could enhance the activation of genes induced by IFN-γ, and then, participate in the consequent processes. IRF-8(-) mice are not only associated with immunodeficiency due to the defects in functions of macrophages, but also can progress to CML-like diseases (22). A study performed by Waight et al. demonstrated the importance of the *BCR-ABL-STAT5-IRF-8 axis.* IRF-8 is the direct target of STAT5 in CML, while silencing of STAT5 can induce the expression level of IRF-8. *BCR-ABL*-mediated IRF-8 inhibition is the result of a direct influence of STAT5 on the promoter of IRF-8. The findings also revealed that the activation of STAT3 could also inhibit the transcription of IRF-8 (30).

IRF-8 is member of IRF family that mainly regulates the growth and differentiation of BMPCs, as well as the activation of functions of macrophages. The expression level of IRF-8 influences the processes of various diseases ranging from infection to leukemia profoundly, and indicates that IRF-8 plays a key role in development of myeloid cells. Further studies reported the exact molecular mechanisms underlying the regulation of BMPCs by IRF-8.

## IL-9

It is well known that different CD4+ T cell subgroups (e.g., Th17 and Th9) and innate immune cells, such as mastocytes and group 2 innate lymphoid cells (ILC2s), could produce IL-9. IL-9 is a pleiotropic cytokine that could activate STAT1, STAT3, and STAT5 to influence the functions of various target cells (e.g., T cells, B cells, mastocytes, and airway epithelial cells). Due to the pleiotropic effects, IL-9 has been proven to participate in diverse diseases, including cancer, autoimmune diseases, and other pathogen-mediated immune regulatory diseases.

### IL-9 Is Produced Primarily by TH9 Cells

Th9 cell is a new subgroup of CD4+ T cells that was originally discovered in 2008, and it was characterized by the secretion of IL-9 (31). In worm models of infection, the development of Th9 cell was detected and was considered as the major endogenous source of IL-9 (32). The co-existence of IL-4 and TGF-β could stimulate juvenile T cells to differentiate into Th9 cells (33). Veldohen et al. demonstrated that TGF-β could promote the transition of Th2 to Th9 cells (34), while Dardalhon et al. pointed out that IL-4 could inhibit the Foxp3 expression in Treg cells (35). IL-9 could also be produced by CD8+ Tc9 cells, Vδ2 T cells, and mastocytes, and it plays a role in anti-tumor immunity (33). Some additional cytokines could amplify the production of IL-9. IL-2 could activate STAT5 that bind to *IL9 gene* to enhance IL-9 expression (36, 37). Recently, the combination of IL-1β and IL-4 has been demonstrated to activate the nuclear factor-κB (NF-κB) to enhance the induction of Th9 cells, even in the absence of TGF-β signal transduction (38, 39). In the majority of solid tumors, IL-9 could directly promote the apoptosis of tumor cells or activate the innate and adaptive anti-tumor immunity.

### Pu.1 and IRF-4 Play Important Roles in TH9 Cell Development and Differentiation

STAT6 and GATA3 are required for the first, second, and fifty generations of Th9 cells. The selective expression of TGF-β-induced pu1 in Th9 cells could restrict the capability of STAT6 and GATA3 in inducing Th2-type cytokines. Gene expression analysis indicated that Th9 cells have evident transcription characteristics (40). In addition, Th2 cells cultured with TGF-β could be transited to Th9 cells (34). Previous studies have shown that transcription factors of ETS family, such as pu1 and IRF4, play important roles in the development of Th9 cells, and were considered as essential transcription factors for the development of Th9 cells (41, 42). Deletion of pu1 could impair the generation of Th9 cells, while the reactions of Th2 cells are normal, indicating that pu1 is a key regulator for Th9 cell differentiation (41). The ectopic expression level of pu1 in Th2 cells triggers the low expression level of Th2-type IL-9, indicating that pu1 could be a switch-on factor for induction of IL-9 in Th9 cells (41, 43).

### The Mode of Action of IL-9 on Tumor and Its Carcinogenic Activity in Lymphoma

IL-9 was initially described as a growth factor secreted by activated helper T cells type 2 (Th2), which exerts the effects through the members of the γc family of cytokines on target cells, affects various immune cells through IL-9R, and plays different functions in immune and inflammatory responses (44–46). The signal transduction mediated by IL-9 mainly depends on the high affinity between IL-9 and IL-9R. IL-9 plays an anti-tumor role in solid tumors, such as melanoma and breast cancer (47–49). While in hematologic tumors, including chronic lymphocytic leukemia (CLL), Hodgkin lymphoma (HL), and DLBCL, it is generally acknowledged that IL-9 promotes tumor progression through the T-lymphocyte growth factor (50–52).

The function of IL-9 in promoting lymphocyte transition is directly mediated by the activation of Janus kinase and phosphorylation of STAT3 and STAT5 signaling pathways (53, 54). Especially, activation of STAT5 has been considered as the key mediator for IL-9-driven proliferation and tumorigenesis (53). In addition, IL-9 could indirectly mediate the immunosuppressive effects on mastocytes and Treg cells in mouse models of lymphoma to inhibit tumor growth (55). The secretion of IL-9 in tumor microenvironment has been considered as a tolerance factor that inhibits adaptive anti-tumor immunity (56).

IL-9 could directly influence the survival of tumor cells (57), or activate mastocytes and recruit DCs to the tumor site, and it indirectly participates in tumor immunity (58, 59). The dysregulation of IL-9 and IL-9R could be detected in the biopsy specimens and serum of patients with HL, anaplastic large cell lymphoma (ALCL) (55), and nasal NK/T cell lymphoma (60, 61). Previous studies have demonstrated that the serum IL-9 level is elevated in patients with B-cell NHL (including some patients with DLBCL). Using a neutralizing antibody to block the binding of IL-9 to IL-9R could significantly inhibit the tumor growth in mouse models of lymphoma (62).

As a lymphocyte growth factor, IL-9 could promote the proliferation and activation of lymphocytes, thereby exerting tumorigenic effects on hematological tumors (63). It was reported that IL-9 could promote the survival and drug resistance of cells in patients with DLBCL, while silencing of *IL-9R gene* could reduce IL-9-induced drug resistance, and thus, IL-9 provides a potential therapeutic target for DLBCL (50). IL-9 has also been demonstrated to enhance the Treg and mastocyte-mediated immunosuppression effects to participate in the pathogeneses of B-cell NHL (55). In addition, IL-9 could stimulate the proliferation of lymphomas and protect them from dexamethasone (DEX)-induced cell apoptosis (64, 65). The carcinogenic activity of IL-9 in lymphomas has already been demonstrated by numerous studies. Serum level of IL-9 in patients with DLBCL is significantly elevated, which is associated with a low serum level of albumin and a high international prognostic index (IPI) (50). *In vitro* studies have shown that IL-9 could upregulate the *P21CIP1 gene* in tumor cells to directly induce the proliferation and inhibit the apoptosis of LY1 and LY8 cells in DLBCL, promote the survival of DLBCL cells, and reduce the sensitivity of tumor cells to chemical substances (50).

Future studies on cell biology and clinical relevance of Th9 could improve our understandings on immune regulation of Th9 cells, and finally lead to the effective treatment of human diseases with such cells involved.

## AP-1

The AP-1 complex is a dimer transcription factor, and human AP-1 family is composed of the homo- and hetero-dimers of Jun (c-Jun, JunB, and JunD), Fos (c-Fos, FosB, FRA1, and Fra2), ATF (ATF2, ATF3/LRF1, B-ATF, JDP1, and JDP2), and MAF (cMaf, MafB, MafA, MAFG/F/K, and Nrl) family members. All these family members possess a highly conservative basic leucine zipper (bZIP) protein binding domain (66, 67), and participate in the basic and inductive transcriptions of several genes containing AP-1 site (68).

### Ap-1 Has Multiple Roles in Inflammation and Tumor Development

AP-1 participates in the regulation of different cellular processes, including proliferation, apoptosis, differentiation, survival, migration, and transition (66, 69–72). All these AP-1 proteins actively participate in the development and progression of tumors. Numerous studies have demonstrated that the AP-1 transcription factor plays a critical role in proliferation, apoptosis, angiogenesis, and oncogene-induced transition and invasion, and is also involved in diverse types of cancer, including breast cancer, ovarian cancer, liver cancer, skin cancer, bone cancer, lung cancer, endometrial cancer, colorectal cancer, and hematological tumors (68).

### Inflammatory Cell Signal Transduction Is Implicated in the Future Development of Cancer

Previous studies have reported that cell signaling of inflammatory is associated with the future development of cancer (73, 74). AP-1 is an important component of inflammatory response (66, 75). In chronic inflammatory diseases, various cytokines and chemokines are recruited at the site of inflammation, and are mainly regulated by ap-1 (fos/jun) and other transcription factors, such as NF-κB, NFATs, and STATs (76, 77). Ap-1 directly binds to the ap-1 binding sequence in promoters, and consequently regulates the expression levels of cytokines (TNF-α, IL-1, IL-2, IFN-γ, and granulocyte-macrophage colony-stimulating factor (GM-CSF)), as well as matrix metalloproteinases (MMPs) at the mRNA synthetic level (66). It has been reported that AP-1 participates in the differentiation of primitive T cells to Th1 and Th2 cells (78, 79).

### Inadequate Activation of AP-1 Is Associated With Immune System Disorders

Jun and Fos could promote DNA synthesis, participate in the generation and lysing capability of CD8 T lymphocytes through the proceeding of cell cycle, contribute to the regulation of cell proliferation and activation, and take part in development and function of lymphocytes (75). In patients receiving hematopoietic stem cell transplantation (HSCT), patients with T cell deficiency are associated with a poorer prognosis; patients treated with allogeneic HSCT are accompanied with downregulated expressions of *c-jun and c-fos gene* in T cells. Such deficiencies may justify insufficient CD4 activation (80–82). These conditions could be recovered over time after transplantation. However, in patients with a poor prognosis, the findings showed that the gene expression decreased time-dependently (82), indicating that deficiency of AP-1 activation was associated with immune system disorders. The immunological reconstitution in patients undergoing transplantation requires the increase of the expression levels of such oncogenes within the first 2 years; otherwise, lethal outcomes are expected.

### Ap-1 and Hematological Malignancies

In hematological malignancies, AP-1 components are involved in CML and AML (83–85), and play important roles in the pathogeneses of lymphomas, HD, and ALCL (86, 87). In AML, c-fos could exert tumor growth inhibitory activity or result in poor outcomes (84, 85). Compared with untreated CML patients, the lymph node mononuclear cells of imatinib-treated CML patients showed AP-1 activation, as well as downregulation of pro-inflammatory cytokines (88), which could be caused by the inactivation of *JunB gene* methylation, indicating that *JunB* could be a tumor suppressor gene (89). Besides, activated AP-1 with robust c-Jun and JunB overexpression was found in all tumor cells of patients with HL (86, 87). C-Jun could be upregulated through autoregulation, while JunB could be upregulated through NF-κB (87). The activated AP-1 could support the proliferation of Hodgkin cells, and inhibit the apoptosis of ALCL cells.

### Activation of AP-1 Complex Is an Important Factor Regulating the Growth of ABC-DLBCL

Activated B cell (ABC)-DLBCL is characterized by poor outcomes, and it is associated with the constitutive activation of NF-κB, controlling and promoting the cell proliferation, survival and gene expression. Similar to NF-κB, AP-1 is regulated by the constitutive activation of the B-cell receptor signaling component caspase recruitment domain-containing membrane-associated guanylate kinase protein-1 (CARMA-1) (90). Compared with germinal center (GC) B cell (GCB)-DLBCL, the ABC-DLBCL cell lines expressed high levels of AP-1 family members, such as c-Jun, JunB, and JunD, which form hetero-dimer with AP-1 family member-activating transcription factors, including ATF2, ATF3, and ATF7. Using the dominant-negative method to inhibit such complexes could induce growth impairments in most ABC-DLBCL cell lines. The individual silencing of c-Jun, ATF2, or ATF3 could reduce the survival rate of cells, confirming that the activation of Jun/atf type AP-1 complex is an important factor regulating the growth of ABC-DLBCL (90).

### IL-21 Promotes DLBCL Cells Proliferation by Upregulating of AP-1

The clinical characteristics of Epstein-Barr virus (EBV)-positive DLBCL indicated that the outcomes of this disease are poorer than EBV-negative DLBCL. It was reported that IL-21 could promote the apoptosis of DLBCL cells. However, studies on IL-21-stimulated EBV-positive DLBCL cells showed that IL-21 could upregulate the phosphorylation of host MYC, AP-1, and STAT3, as well as the expression of viral LMP-1 protein, thereby promoting the proliferation of DLBCL cells (91).

## Potential Regulatory Relationship Between IL-9, IRF8, and AP-1 Families

The above-mentioned outcomes indicated the respective effects and molecular mechanisms of IRF-8, IL-9, and AP-1, as well as the associations with DLBCL or cancer cells. When we reviewed the literature, we found that these molecules are closely related to some cytokines or transcription factors directly or indirectly, which are summarized as follows.

### IRF-8 and AP-1 in the Production of IFN-γ

**Figure 1:**
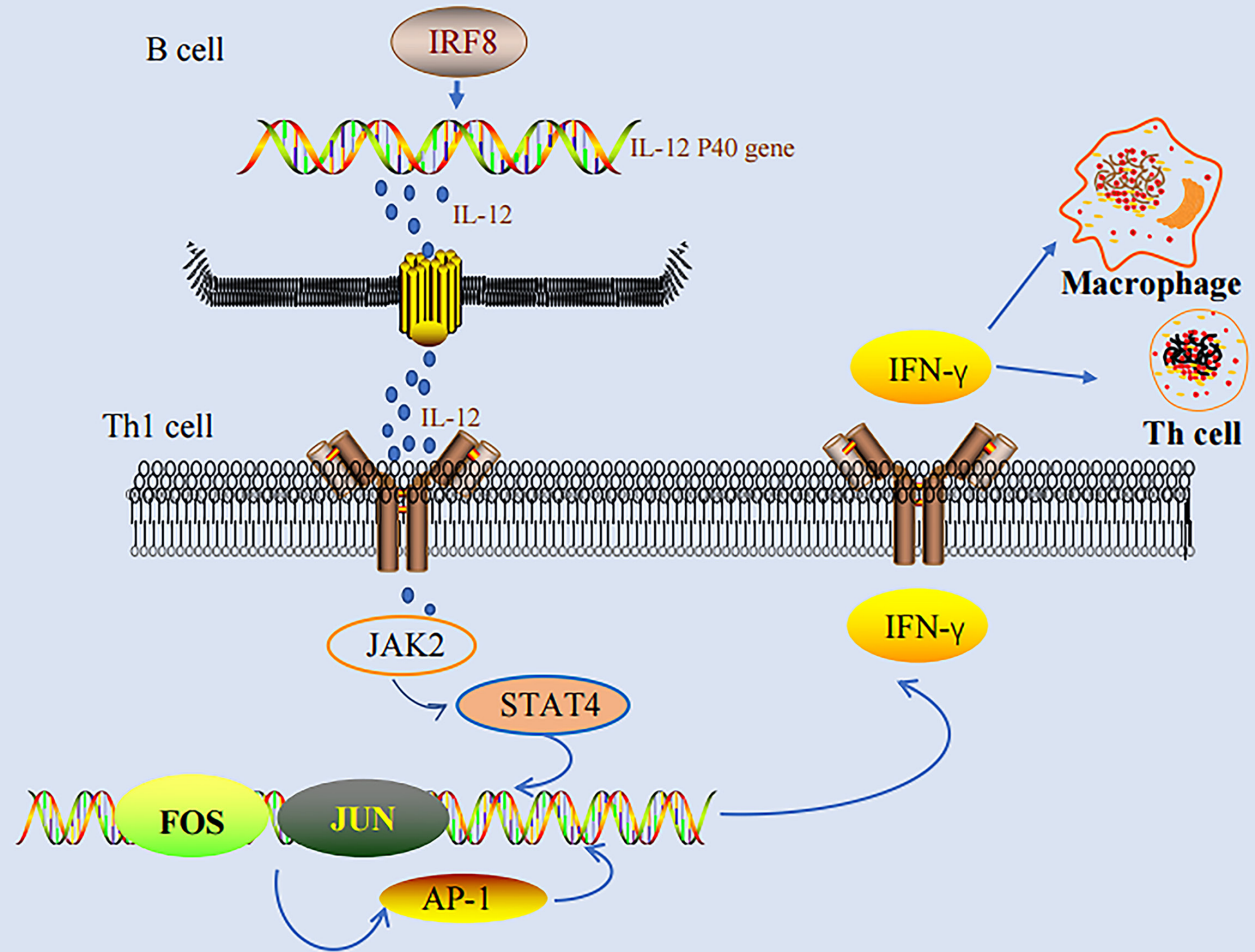
IRF-8-IL-12-IFN-γ pathway and AP-1-IFN-γ pathway.

IFN-γ stimulates various genes in macrophage to activate the activities of macrophages, including phagocytic effects, anti-bacterial effects, cytokine production, and antigen presentation, which are the basis for the defense of host against infection (17). IRF-8 could enhance the activation of IFN-γ-inducing genes, and therefore, participate in the consequent processes of infection. IRF-8 regulates the induction of *IL-12 p40* and substantially influences the differentiation of NK and Th1 cells in an IFN-γ-dependent manner. IFN-γ could not induce Th1-mediated responses in IRF-8 (-) mice (22, 28, 29). On the other hand, AP-1 binds to AP-1 binding sequences directly, thereby regulating the expression of IFN-γ at the mRNA synthetic level (66). Studies have also demonstrated that AP-1 participates in the differentiation of primitive T cells to Th1 and Th2 cells (78, 79). We found that both IRF-8 and AP-1 play important roles in IFN-γ production. In addition, both IRF-8 and AP-1 play important roles in the development and progression of tumors. However, additional studies are needed to investigate the exact role of AP-1 in IRF-8-regulated Th1 responses (**Figure 1**).

### The Role of IRF-8 in IL-9 Production and Other Regulatory Pathways of IRF-8

**Figure 2:**
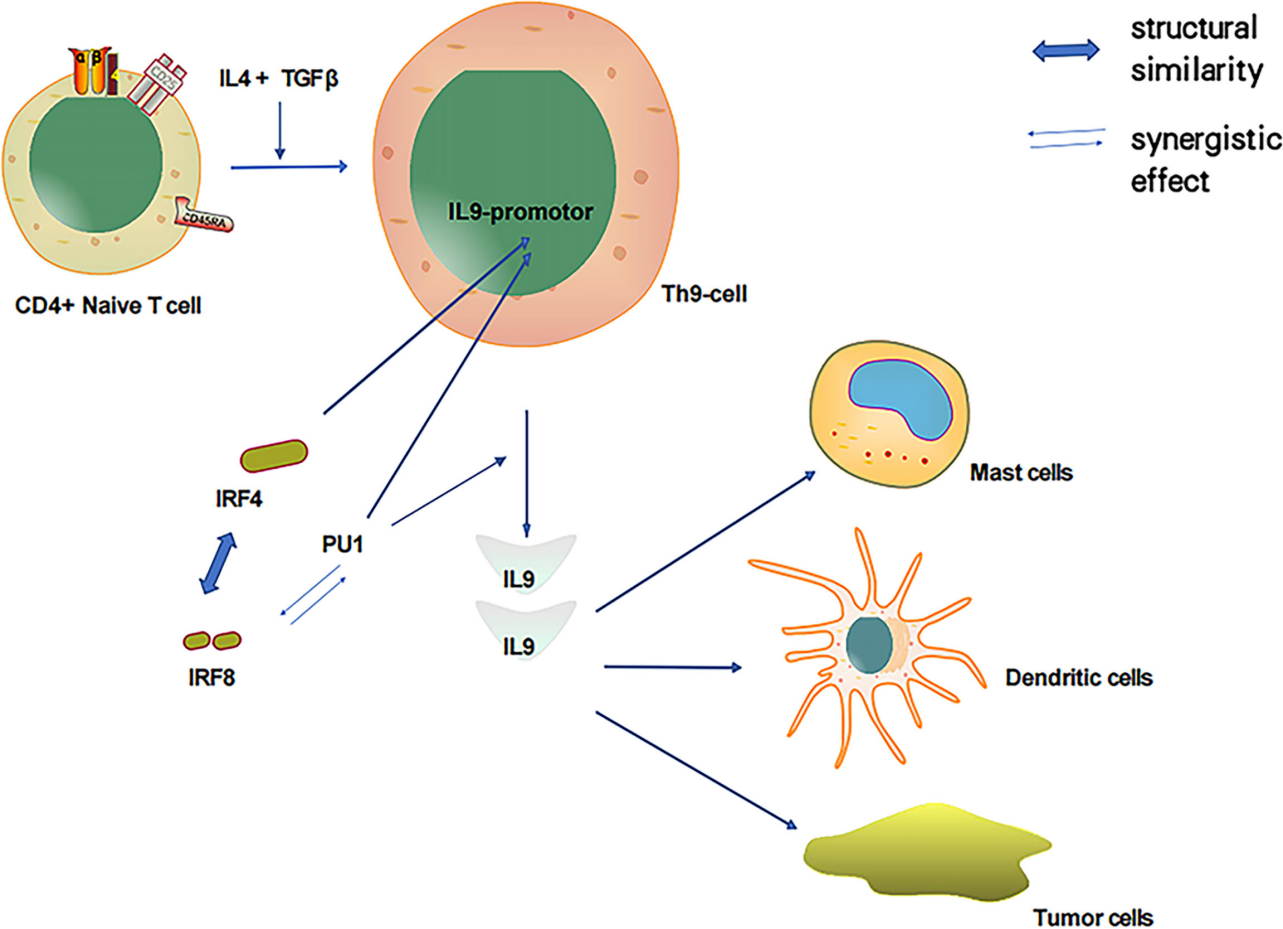
IL-4 and TGFβ induced T cells to produce IL-9 and relationship between important transcription factors PU1, IRF-4 and IRF-8.

As discussed, both PU.1 and IRF-4 play pivotal roles in Th9 cell development as essential transcription factors. In particular, PU.1 is a key regulator of Th9 cell development (41, 42). Of the IRF family members, IRF-4 has the highest amino acid sequence similarity to IRF-8; thus, IRF-8 could also play a notable role in Th9 cell development. IRF-8 is essential for Th9 differentiation *in vitro* and *in vivo*. IRF-8 functions as part of a transcription factor complex composed of IRF-8, IRF-4, PU.1, and BATF, which binds to DNA and promotes IL-9 transcription (92). PU.1 and IRF-4 regulate a group of overlapping target promoters by interacting with common chaperons (such as PU.1 and E47) (4–7). The direct interaction between the PU.1 protein and IRF-8 substantially enhances the DNA binding activity of IRF-8 (11, 16). Additionally, PU.1 and IRF-8 have a synergistic effect that activates the promoters of myeloid-specific genes. Therefore, PU.1 is closely associated with IRF-8 and IL-9, and IRF-8 could participate in the induction of IL-9 produced by Th9 cells (**Figure 2**).

**Figure 3:**
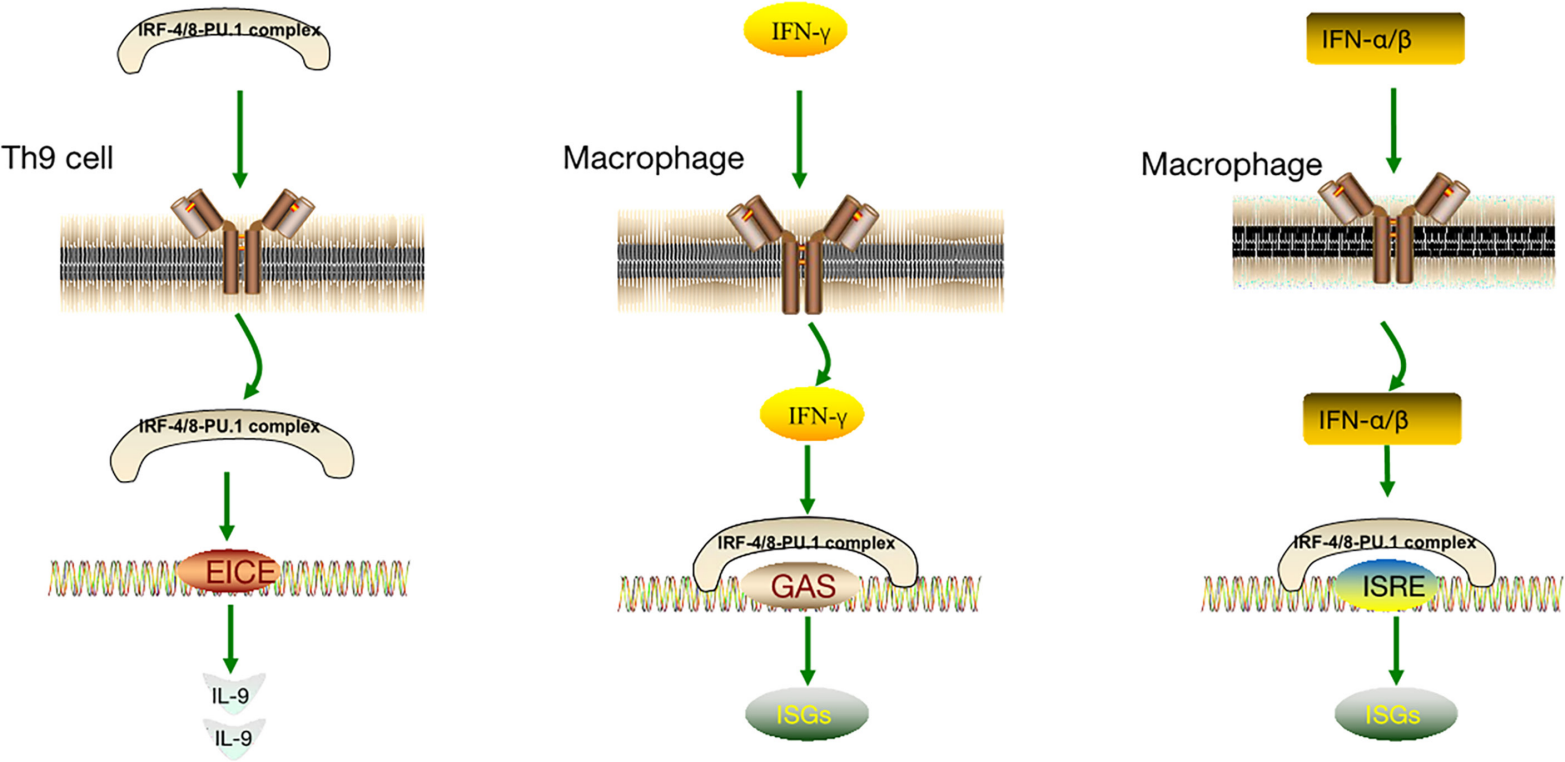
IRF-8-IL-9 regulatory pathway and other IRF-8 regulatory pathways.

The IRF-4/8-PU.1 complex is also involved in the regulation of other pathways (**Figure 3**). Some IFN-induced genes carry IFN- γ-activated site (GAS) elements that contain binding sites for the IRF-8-PU.1 complex. The IRF-8-PU.1 complex participates in GAS-mediated transcription and amplifies the expression of STAT1-activated IFN-γ response genes in macrophages (93). Similarly, some IFN-α/β–induced genes carry IFN-stimulated response elements (ISREs) containing IRF-8-PU.1 binding sites. IRF-8-PU.1 participates in ISRE-mediated transcription, enhancing the transcription induced by IFN-stimulated gene factor 3 (ISGF3) in macrophages, which contribute to viral infection resistance by producing ISG family proteins (93). By comparing these pathways (**Table 1**), we concluded in the two pathways above, IRF-4/8-PU.1 complex plays a protective role in protecting against infection, but could not explain the effect of the two regulations on tumors and DLBCL. It is more meaningful to study the irF-8/IL-9 regulatory pathway.

**Table 1:** IRF-8-IL-9 regulatory pathway and other IRF-8 regulatory pathways.

	IRF-4/8-PU.1 complex	IRF-4/8-PU.1 complex	IRF-4/8-PU.1 complex
Target cells	TH9 cell	Macrophages	Macrophages
Binding site	ETS/IRF composite element (EICE)	IFNγ activated site element (GAS)	IFN stimulates response elements (ISREs)
Function	Binds to DNA and promotes IL-9 transcription	Amplification of STAT1-activated IFN-γ response gene expression in macrophages	Transcription enhancement induced by IFN-α/β – stimulated gene factor 3 (ISGF3).
Production	IL-9	ISGs	ISGs
Results	Stimulates the proliferation of lymphoma cells	Resistance to viral infection	Resistance to viral infection

### The Relationship Between AP-1, IL-9 and NF-KB Pathway, and Chronic Inflammation Promote the Development of DLBCL

**Figure 4:**
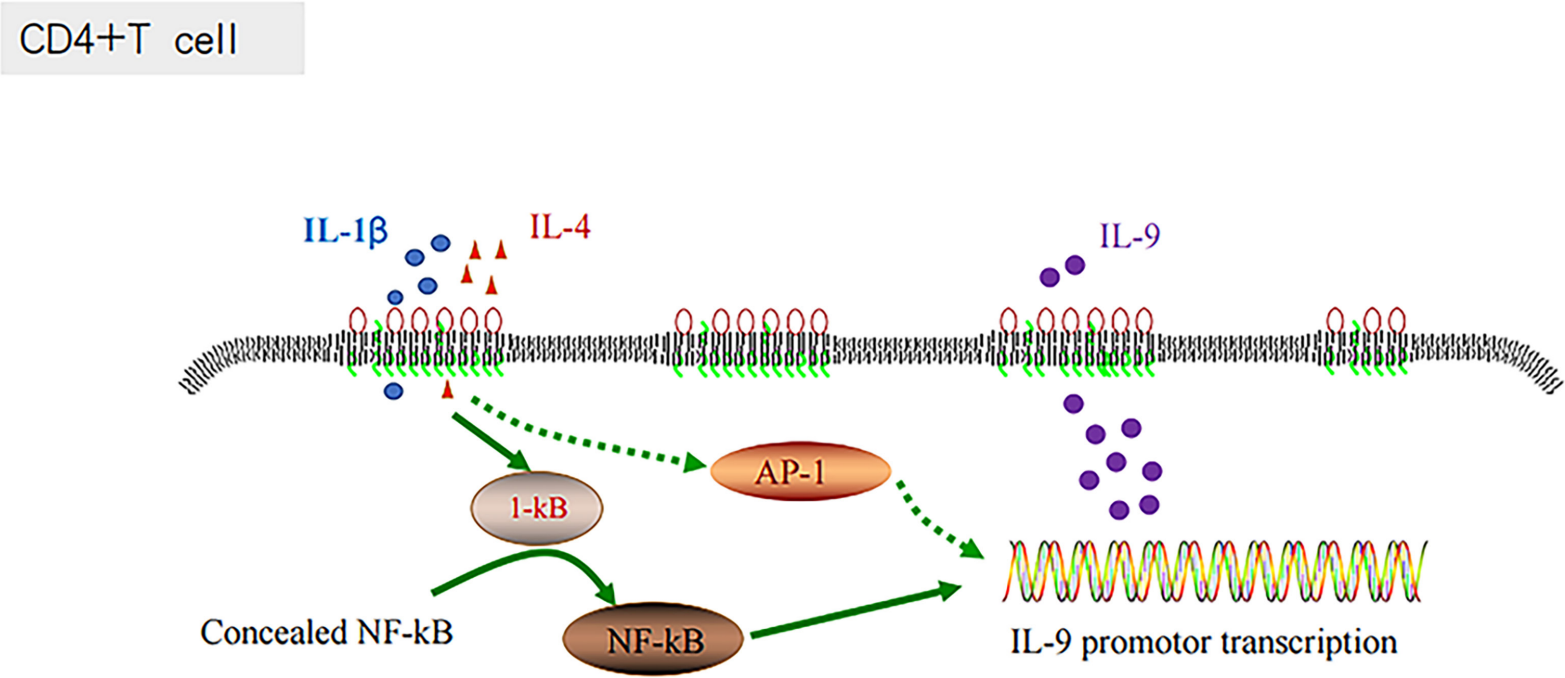
IL-1 β and IL-4 induce T cells to produce IL-9 through the NF-KB pathway.

The inflammatory signaling pathways are associated with the development of diverse types of cancer (73, 74). AP-1 is an important component of inflammatory responses (66, 75). In chronic inflammatory diseases, various cytokines and chemokines are recruited at the site of inflammation, which are mainly regulated by ap-1 (fos/jun) and other transcription factors, including NF-κB, NFATs, and STATs (76, 77). ABC-DLBCL is a molecular subtype characterized by poor outcomes, and it is associated with the constitutive activation of NF-κB, controlling and promoting the cell proliferation, survival and gene expression (90). The combination of IL-1β and IL-4 has been proven to activate the NF-κB signaling pathway and enhance their inductive effects on Th9 cells, and consequently expand the production of IL-9 (38, 39). Similar to NF-κB, AP-1 could also be regulated by the constitutive activation of the B-cell receptor signaling component CARMA-1 (90). There could be some associations between Th9 cells and AP-1, and IL-1β and IL-4, which could regulate IL-9 secretion through AP-1 (**Figure 4**).

The development of DLBCL is closely associated with the activity of numerous inflammatory mediators and cells. Inflammatory mediators affect tumor occurrence and development by directly or indirectly affecting the behavior of immune cells, whereas inflammatory cells promote tumor progression by producing cytokines. For example, mast cells and ILC2s both produce the cytokine IL-9. IL-2 enhances IL-9 expression by activating STAT5 (36, 37). IL-9, however, stimulates the proliferation of lymphoma cells and protects them from the effects of dexamethasone-induced apoptosis (64). In addition, AP-1 is an important component of the inflammatory response and is involved in the differentiation of primitive T cells into Th1 and Th2 cells (66, 75, 78, 79). AP-1 is involved in the control of cell proliferation and activation and the development and function of lymphocytes (75). For example, IL-21 promotes the proliferation of DLBCL cells by upregulating the phosphorylation of host MYC, AP-1, and STAT3 and the expression of viral LMP-1 protein (91).

IL-9 and AP-1 play important roles in chronic inflammation, which affects the progression of DLBCL. IL-1β and IL-4 enhance TH9 cell induction by activating the NF-κB pathway, inducing IL-9 production (38, 39). AP-1 complex activation is an important factor controlling the growth of ABC-DLBCL. AP-1 complex activation is marked by constitutive activation of the transcription factor NF-κB, which controls the expression of genes promoting cell survival and proliferation (90). ABC-DLBCL is a molecular subtype characterized by adverse outcomes. Therefore, the NF-κB pathway plays a significant role in the influence of IL-9 and AP-1 on DLBCL progression and prognosis.

## Conclusion and Perspective

In this review, we summarized the mechanisms of action of IRF-8, IL-9, and AP-1 and discussed how these factors promote tumor development, with a particular focus on DLBCL. We also described the interactions and synergistic effects involved in DLBCL development. IRF-8 is essential for *in vitro* and *in vivo* Th9 cell differentiation and plays an important role in DLBCL development. NF-κB plays a significant role in both IL-9 production and the AP-1 inflammatory signaling pathway and may be a hub for AP-1 and IL-9 connections. This review suggests new directions for further research regarding DLBCL-specific molecular markers and signal transduction pathways, DLBCL pathogenesis, and targeted therapies.

## Author Contributions

MC carried out the primary literature search, drafted and revised the manuscript, and participated in discussions. NC revised and edited the final version of the manuscript. All authors read and approved the final manuscript.

## Funding

The present study was partly supported by the Clinical Medicine Science and Technology Innovation Program, China (no.202019055).

## Conflict of Interest

The authors declare that the research was conducted in the absence of any commercial or financial relationships that could be construed as a potential conflict of interest.

## Publisher’s Note

All claims expressed in this article are solely those of the authors and do not necessarily represent those of their affiliated organizations, or those of the publisher, the editors and the reviewers. Any product that may be evaluated in this article, or claim that may be made by its manufacturer, is not guaranteed or endorsed by the publisher.
